# Alveolar Bone Grafting in Unilateral Cleft Lip and Palate: Impact of Timing on Palatal Shape

**DOI:** 10.3390/jcm12247519

**Published:** 2023-12-05

**Authors:** Andrzej Brudnicki, Tereza Petrova, Ivana Dubovska, Anne Marie Kuijpers-Jagtman, Yijin Ren, Piotr S. Fudalej

**Affiliations:** 1 Department of Pediatric Surgery, Institute of Mother and Child, 01-211 Warsaw, Poland; andrzej.brudnicki@imid.med.pl; 2 Department of Orthodontics and Cleft Anomalies, 3rd Medical Faculty, Faculty Hospital Royal Vineard, Dental Clinic, Charles University, 11636 Prague, Czech Republic; trz.petr@gmail.com; 3 Institute of Dentistry and Oral Sciences, Faculty of Medicine and Dentistry, Palacký University Olomouc, 77900 Olomouc, Czech Republicpiotr.fudalej@uj.edu.pl (P.S.F.); 4 Department of Orthodontics, University Medical Center Groningen, University of Groningen, 9713 GZ Groningen, The Netherlands; y.ren@umcg.nl; 5 Department of Orthodontics and Dentofacial Orthopedics, School of Dental Medicine, Medical Faculty, University of Bern, 3010 Bern, Switzerland; 6 Faculty of Dentistry, Universitas Indonesia, Campus Salemba, Jakarta 10430, Indonesia; 7 Department of Orthodontics, Jagiellonian University in Cracow, 31-007 Krakow, Poland

**Keywords:** cleft palate, palatal shape, alveolar bone grafting, early secondary bone grafting

## Abstract

Alveolar bone grafting (ABG) is a critical surgical intervention in patients with a cleft of the alveolus, aimed at reconstructing the alveolar ridge to facilitate proper eruption, periodontal support, and alignment of adjacent permanent teeth. The optimal timing for ABG remains debated, with late secondary ABG between the ages of 9 and 11 being widely adopted. This study compared the palatal shapes of 28 children at a mean age of 9.5 years (SD = 0.7) who underwent early secondary ABG at a mean age of 2.1 years (SD = 0.6) or 33 children at a mean age of 10.8 years (SD = 1.5) who underwent late secondary ABG at a mean age of 8.6 years (SD = 1.3) to 60 non-cleft controls at a mean age of 8.6 years (SD = 1.2). The palatal shapes were captured with 239 landmarks digitized on the palate on a digital model. Utilizing geometric morphometric methods, i.e., generalized Procrustes superimpositions, principal component analysis, and permutation tests, we assessed the impact of ABG timing on palatal morphology. The first five principal components (PCs) explained 64.1% of the total shape variability: PC1 = 26.1%; PC2 = 12%; PC3 = 11.9%; PC4 = 7.8%; and PC5 = 6.4%. The Procrustes distance between both cleft groups and the control group was more than twice as large as the Procrustes distance between the early ABG and late ABG groups. Nonetheless, all intergroup differences were statistically significant. Our findings suggest that early ABG has a limited negative effect on palatal shape, providing comparable outcomes to late ABG. The study highlights the potential suitability of early ABG, challenging conventional practices and encouraging further exploration into its long-term effects on maxillary growth.

## 1. Introduction

Alveolar bone grafting (ABG) is a surgical procedure performed to reconstruct the alveolar ridge in patients with a cleft of the alveolus. During ABG, bone is harvested from another part of the body, such as the hip or mandibular symphysis, and then transplanted to the cleft area. The grafted bone plays a crucial role in facilitating the proper eruption, periodontal support, and alignment of permanent teeth adjacent to the cleft. By filling the gap and creating a supportive structure, ABG helps stabilize the alveolar ridge, preventing its collapse or deformity. Furthermore, it supports the alar base and contributes to the formation of a more intact and functional oral cavity, which positively impacts speech clarity and articulation.

The youngest age at which ABG has been performed is before or along with the primary closure of the cleft palate. This timing is defined as primary ABG. However, detrimental effects on craniofacial growth have been reported and the procedure has practically been abandoned worldwide [1,2]. When ABG is carried out after the cleft lip and palate have been repaired, it is defined as secondary ABG. The timing generally assumed as the optimal timing for ABG is between the ages of 9 and 11 years, when the root of the permanent canine at the cleft side has reached between 50 and 75% of its final length [3,4,5,6,7]. This approach, called late secondary ABG, has proven to be more successful for subsequent maxillary growth compared to the previously used primary bone grafting [8]. This timing has been widely adopted ever since, despite its not being based on scientific evidence from prospective clinical studies comparing different timings. In fact, the surgical technique of primary ABG—usually performed before surgical repair of the hard palate—requires extensive preparation in the vulnerable region of the vomer, while the secondary bone grafting procedure appears to be less extensive. However, recent longitudinal studies showed that secondary ABG induces the same, very small but registerable maxillary growth inhibition regardless of its timing [9].

The vast majority of cleft teams worldwide tend to follow the conventional timing for late secondary ABG. Only a few teams have implemented early secondary ABG or equivalent procedures in a patient’s early life. For example, a Canadian cleft center in Halifax [10,11,12] has performed grafting at an earlier stage, specifically before or during the eruption of the lateral incisors. This early secondary ABG, typically conducted around 6 years of age, has demonstrated positive effects on periodontal health and provides adequate bony support for permanent teeth adjacent to the cleft, ensuring sufficient bone for future dental interventions. A cleft team in Warsaw (Poland) has performed ABG even earlier, typically between 2 and 3 years of age in patients with unilateral cleft lip and palate (UCLP) [13]. The rationale behind this approach is to benefit from early closure of oro-nasal fistulas and to create better support for the movement and positioning of the tongue and lips during speech development, potentially leading to improved speech articulation. Another example is the cleft team in Milano (Italy) that has performed an equivalent procedure to ABG, called early secondary gingivoalveoloplasty, simultaneously with hard palate repair at around 3 years of age [14,15,16].

Generally, poorer surgical outcomes result in increased palatal scar tissue and a worse prognosis for maxillary growth [17,18,19]. Therefore, assessing palatal morphology is crucial as it reflects the surgical outcomes in the region, including primary repair of the cleft palate and subsequent secondary surgeries (e.g., ABG). This fundamental assumption forms, for example, the basis of the EUROCRAN index, which evaluates not only the occlusal relationship but also the palatal morphology in patients with unilateral cleft lip and palate [20]. When ABG is performed early, such as by the Warsaw cleft team, it may have a significant long-term effect on the shape of the palate. Therefore, the objective of this study was to compare palatal shape in 8–11-year-olds who underwent early secondary ABG or late secondary ABG, and non-cleft controls. The research hypothesis (H_R_) was that all groups would exhibit differences in palatal morphology.

## 2. Material and Methods

This research was carried out as a retrospective observational study comprising three groups, one of which consisted of non-cleft controls. The study received approval from the institutional Bioethics Committee under reference #21/2013. It is essential to note that the surgeons who conducted the primary cleft lip and palate repairs or ABG were not engaged in any aspect of the evaluation outlined in the study.

### 2.1. Participants

Palatal morphology was evaluated in three distinct groups of children: those with complete unilateral cleft lip, alveolus, and palate (CUCLAP), with variations in the timing of alveolar bone grafting (ABG), and a control group of children without any cleft deformity.

The CUCLAP participants underwent one-stage closure of the complete cleft at the Institute of Mother and Child in Warsaw, Poland, as described in a previous study [21]. ABG was conducted on these subjects at different time points, categorized as early (E-ABG) and late (L-ABG). The E-ABG group consisted of individuals selected from a group of 52 consecutive patients who underwent cleft repair between July 1999 and June 2006. Plaster models were available for assessment at an average age of 9.5 years (SD = 0.7), while the remaining 24 subjects in this series either did not possess plaster models or had models of insufficient quality. In the case of the L-ABG group, participants were chosen at random from a series of 61 consecutive patients who had undergone cleft repair between 1993 and 1996.

Both CUCLAP groups had specific inclusion criteria, including non-syndromic status, completion of all surgical treatments at the Institute of Mother and Child (IMC), and the availability of high-quality dental casts taken at approximately 10 years of age. The eligibility of cases in these groups was determined by clinicians based on comprehensive diagnostic information obtained from medical records.

The control group, on the other hand, was drawn from children seeking orthodontic consultation and was selected based on the following inclusion/exclusion criteria: good health, Class I malocclusion, absence of cross-bites, no history of prior orthodontic treatment, no signs of multiple and/or advanced caries, no tooth agenesis or observable supernumerary teeth, and no cleft lip and/or palate or other congenital syndromes within craniofacial structures.

### 2.2. Treatment Protocol

None of the patients received presurgical (infant) orthopedic treatment. The one-stage primary cleft repair involved palatoplasty for both the hard and soft palate and cheiloplasty, all conducted in a single operation, usually when the patients were between 6 and 9 months old. ABG was performed using a cortico-cancellous bone block harvested from the anterior iliac crest, firmly inserted between the bony edges of the alveolar cleft with the cortical lamina facing toward the labial side. The defect space was filled with cancellous chips to ensure no empty spaces remained, and minimal elevation of the palatal periosteum was performed before covering the graft with gingival mucoperiosteal flaps. No bone fragment fixation devices were utilized. Oronasal fistulas, if present, were closed at the same time the bone grafting procedure was performed. The surgical techniques for primary one-stage cleft repair and early and late secondary ABG remained consistent, regardless of the patient’s age or the surgeon performing the operation. All cleft surgeries took place at the IMC and were carried out by a team of five surgeons. The one-stage repairs for UCLP were conducted by the three most experienced surgeons.

Orthodontic treatment involved maxillary expansion to correct posterior cross-bites, typically using removable appliances such as Schwarz’s plates for the majority of patients. Maxillary expansion was typically initiated at a young age, usually before the age of 6. Following active expansion, the removable plates were retained to maintain the stability of the expanded maxilla until alveolar bone grafting was performed. On occasion, quad-helix expanders were used. Fixed appliances were employed only if there was a need to correct central incisor malalignment during this phase. It is important to note that facial masks or skeletal anchorage were not incorporated into the treatment.

### 2.3. Evaluation Methods

**Figure 1:**
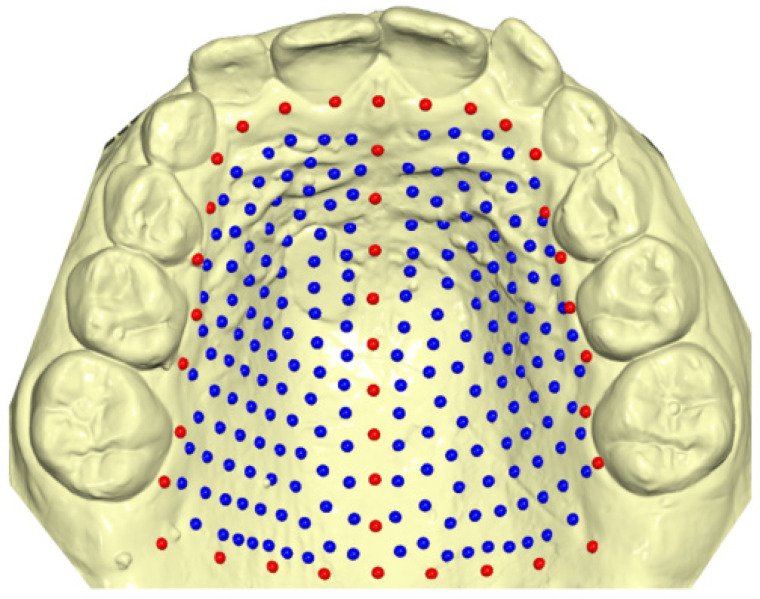
Graphical representation of fixed landmarks (red) and semi-landmarks (blue) drawn on the palatal surface of digital casts.

Plaster casts of the maxilla were digitized using the Trios 3 intraoral scanner (3Shape A/S, Copenhagen, Denmark), and saved as STL files. In cases where the cleft was situated on the right side, the digital scan was horizontally mirrored to ensure that the cleft was consistently on the left side for all subjects. A total of 239 landmarks were identified on each digital model using the Viewbox 4 program (version 4, dHAL software, Kifissia, Greece) (see Figure 1).

Initially, 39 of these landmarks, referred to as “fixed landmarks,” were manually placed on the palate. Among these fixed landmarks were 9 points representing the midsagittal line, 21 points outlining the dental arch, passing apically to the gingival sulci of each tooth, and 9 points defining a posterior curve that extended from the distal aspect of the first permanent molars, perpendicular to the midsagittal line. The remaining landmarks, designated as “semi-landmarks,” were automatically positioned uniformly on the palatal surface within the boundaries defined by the fixed landmarks. Subsequently, these semi-landmarks were adjusted to minimize bending energy, projected back onto the palatal surface, and further adjusted through sliding. This sliding–projecting process was iterated three times, ensuring the homologous positioning of all landmarks across the subjects.

Digitization of the maxillary models was carried out by the same operator. At the time of evaluation, the operator was blinded to the type of intervention (E-ABG vs. L-ABG) for each subject.

### 2.4. Statistical Analysis

Geometric morphometric methods were used for subsequent analysis [22,23]. The homologous landmark configurations underwent generalized Procrustes superimposition. Subsequently, the Procrustes-aligned landmark coordinates were subjected to principal component analysis (PCA), a technique for reducing the dimensionality of data while retaining the most critical information. In this research, PCA was employed to explore the primary patterns of variation in palatal shape. To ascertain the number of principal components containing meaningful shape information, the broken-stick criterion was applied. Differences between the various groups and between males and females were assessed using permutation tests involving 10,000 permutations. For statistical significance, *p*-values below 0.05 were considered.

To assess the method’s reliability, the same observer re-digitized 20 randomly selected maxillary models with an interval of at least 1 month between digitizations. The error was quantified as the Procrustes distance between the repeated digitizations, relative to the total variance in shape in the sample.

## 3. Results

### 3.1. Demographic Data and Method Error

The demographic characteristics of the sample are presented in Table 1. The early ABG (E-ABG) group consisted of 28 children (21 boys and 7 girls). ABG was performed at a mean age of 2.1 years (SD = 0.6) in this group. The late ABG (L-ABG) group comprised 33 children (19 boys and 14 girls). In the L-ABG group, in all patients, bone grafting was performed after the age of 7 years (mean age 8.6 yrs., SD 1.3). The mean age during the evaluation was 9.5 (SD = 0.7) years in the E-ABG group and 10.8 (SD = 1.5) in the L-ABG group. The control group comprised 60 children (25 boys and 35 girls) at a mean age of 8.6 (SD = 1.2) years. As palatal shape was comparable between girls and boys (see below), regardless of the presence of a cleft, the sexes were combined in the analysis. The mean error of the method accounted for 10.8 percent of the total shape variance.

### 3.2. Procrustes Superimposition and PCA

**Figure 2:**
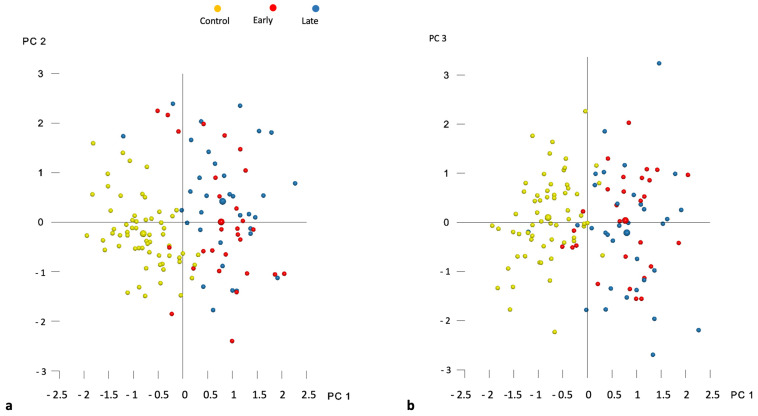
Distribution of individual subjects (smaller circles) and group averages (larger circles) in the shape space described by (**a**) principal components 1 and 2 (PC1 and PC2) and (**b**) principal components 1 and 3 (PC1 and PC3).

**Figure 3:**
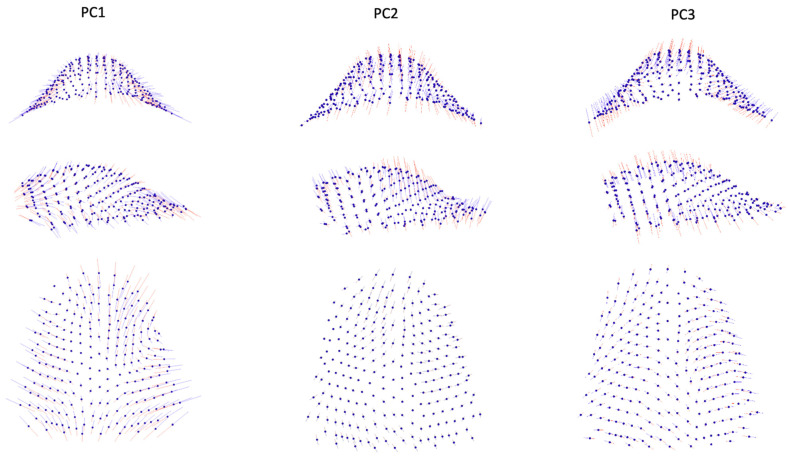
Graphic depiction of the first three principal components (PCs) of the palate from the three views. Red lines: −3 standard deviation; blue lines: +3 standard deviation.

According to the broken-stick criterion, it was determined that the initial twenty-two principal components (PCs) effectively captured significant variations in shape, collectively accounting for 92.9% of the overall shape variability (Table 2). Among these, the first five PCs contributed to at least 5% of the total shape variability, with the breakdown as follows: PC1 = 26.1%, PC2 = 12%, PC3 = 11.9%, PC4 = 7.8%, and PC5 = 6.4%, totaling 64.1%. The distribution of individual subjects in shape space is visually depicted in Figure 2a,b. Additionally, Figure 3 illustrates that PC1 primarily characterizes morphological variations in palate width and length, displaying noticeable patterns of wide and short or narrow and long. PC1 also reveals variations related to the side of the cleft. PC2 predominantly reflects variations in palatal height, while PC3 indicates cleft-side-related variations across all three dimensions.

### 3.3. Intergroup Differences

**Figure 4:**
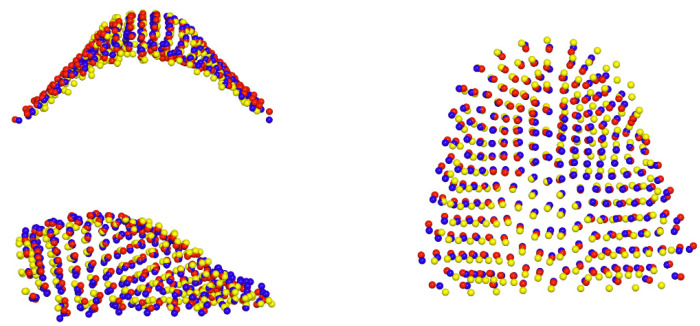
Graphic depiction of superimposed consensus shapes of the control (yellow spheres), early ABG (red spheres), and late ABG (blue spheres) groups from the three views.

Table 3 presents the differences in shape space between the groups. The Procrustes distance between the control group and both cleft groups—E-ABG and L-ABG—was more than twice as large as the Procrustes distance between the E-ABG and L-ABG groups. Nonetheless, all intergroup differences were statistically significant. Figure 2a,b illustrate that the most significant differences between the control group and both cleft groups were observed along the PC1 axis, with participants without a cleft displaying wider and longer palates compared to participants from the E-ABG and L-ABG groups. Conversely, the E-ABG and L-ABG groups exhibited prominent differences primarily along the PC2 and PC3 axes. The superimposition of consensus shapes of both cleft groups (Figure 4) suggests that early ABG resulted in a slightly narrower anterior palate, particularly in the former alveolar cleft region.

## 4. Discussion

Alveolar bone grafting, like any surgical procedure conducted in a growing child, has the potential to affect the natural growth and development of the maxilla and its surrounding structures [24,25]. To mitigate this interference, it is generally recommended to postpone the grafting until most maxillary growth has occurred [26,27,28]. As a result, most cleft centers perform ABG when the child’s permanent canine at the cleft side starts to erupt, which typically happens between the ages of 9 and 11 years. However, this timing of ABG can lead to inadequate bone support for the lateral incisor, and its loss in the worst-case scenario. The objective of this study was to assess the impact of alveolar bone grafting performed between 2 and 4 years of age on maxillary growth by comparing the palatal morphology of children who underwent early or late ABG, and non-cleft controls.

Our findings indicate that the impact of early ABG on palatal morphology is limited and does not seem to have significant clinical implications. Theoretically, the formation of scar tissue on the palatal surface in areas sensitive to growth could impact the shape of the palate. This is because scar tissue, characterized by densely packed and disorganized collagen fibers, is less elastic and flexible than normal tissue. The structural and compositional differences in scar tissue have the potential to impede growth [19], contributing to alterations in palatal shape. However, long-term observations of maxillary growth [9] or alveolar bone volume [21] subsequent to bone grafting at various timings have revealed that the suppressive impact of this surgical procedure is not enduring, which contrasts with what one might anticipate from scar formation. Instead, the inhibitory effect is active for a finite period, beyond which bone tissue appears to return to its normal growth patterns. The practical implication here is that bone grafting should be avoided shortly before or during phases of rapid growth, such as the prepubertal growth spurt or before the age of three. The growth trajectory from the 3^rd^ year of age until the onset of the prepubertal growth spurt remains relatively stable and is not correlated with the eruption of canines. In our study, patients in the early ABG group had ABG performed at 2 years and were evaluated approximately 7.5 years later. Conversely, patients in the late ABG group underwent ABG at an average of 8.7 years and were evaluated approximately 2 years later. A comparable evaluation at maturity after growth cessation would likely yield more conclusive findings.

**Figure 5:**
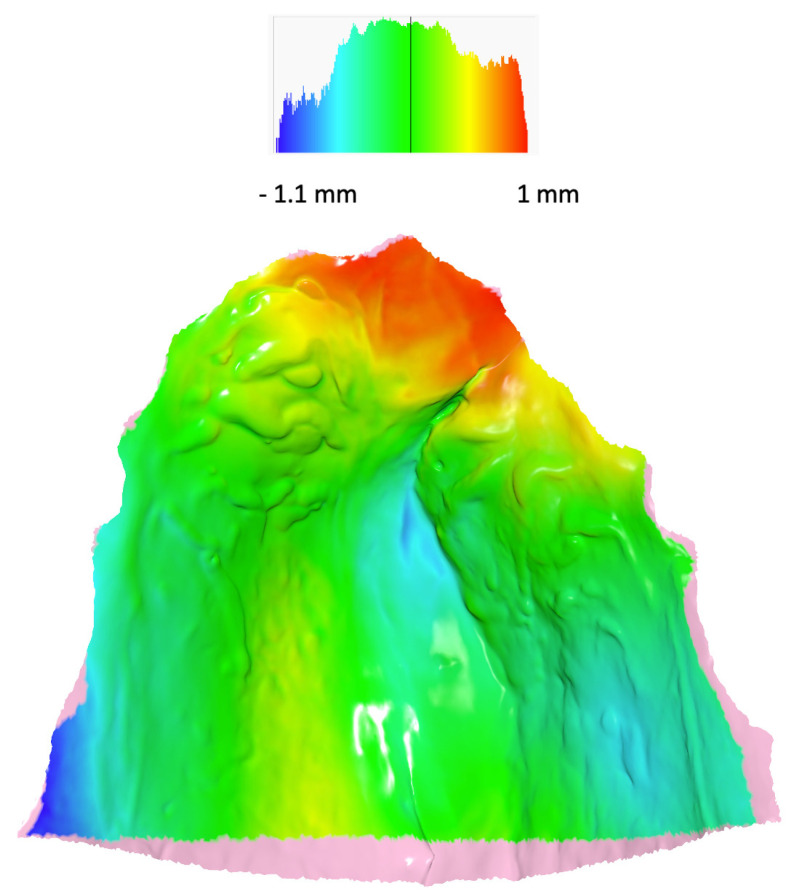
Heat map with the consensus shape of the late ABG group used as reference. Colors describe which parts of the palate of the consensus shape of the early ABG group are in front of the reference (red, yellow), close to the reference (green), or behind the reference (blue). The pink color denotes overhang regions for which a heat map was not calculated.

When compared to children from the late ABG group, the palates of the early ABG group showed remarkable similarity. Superimposition of consensus shapes from both groups (Figure 5) revealed some constriction in the region of the bone graft after early ABG, but the magnitude of this effect was relatively small. Our present results align with our previous studies, in which we observed that early ABG led to a slightly greater collapse of the lesser segment compared to bone grafting performed between 9 and 12 years of age [29]. However, this collapse had minimal consequences for craniofacial growth [13]. Unfortunately, we were unable to compare our findings with results from other studies because early ABG at a similar timing to ours is rarely carried out by cleft centers worldwide, and no reports have been published yet [30].

**Figure 6:**
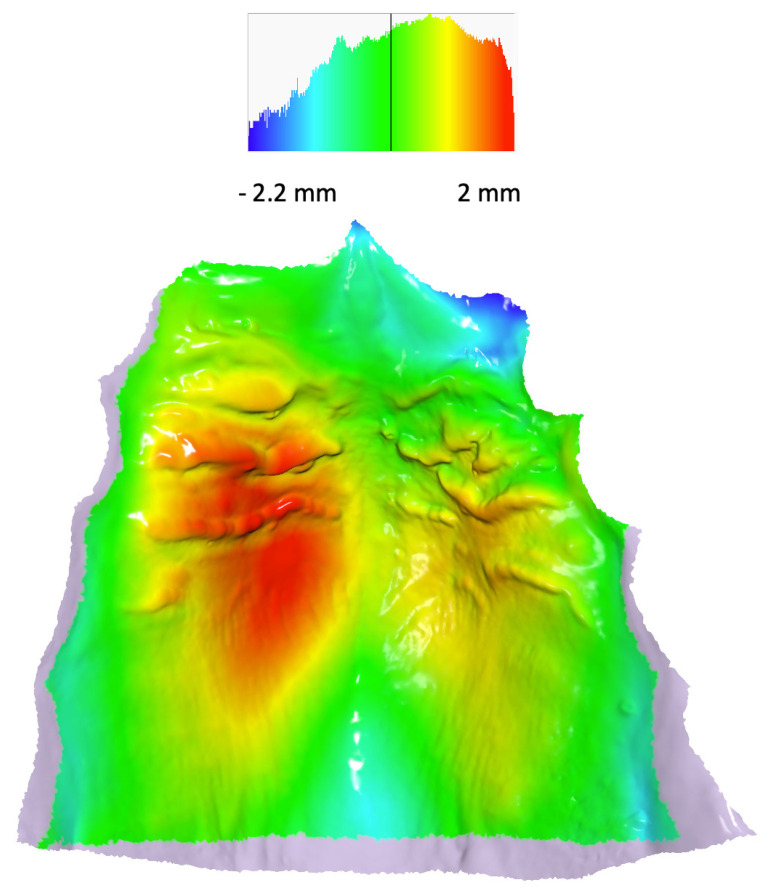
Heat map with the consensus shape of the control group used as reference. Colors describe which parts of the palate of the consensus shape of the pooled early and late ABG groups are in front of the reference (red, yellow), close to the reference (green), or behind the reference (blue). The pink color denotes overhang regions for which a heat map was not calculated.

In contrast to a minor disparity in palatal shape observed between subjects who underwent early and late ABG, there was a significantly greater distinction in palatal morphology between children without a cleft and those with a cleft. Heat maps (Figure 5 and Figure 6) revealed that the dissimilarity between the average palatal shapes of the control group and the combined E-ABG and L-ABG groups ranged from approximately −2.2 to 2 mm, while the dissimilarity between the average palatal shapes of the E-ABG and L-ABG groups was half that size, ranging from −1.1 to 1 mm. Furthermore, the disparity in shape between children with and without a cleft was predominantly observed along the PC1 axis, which represented variation in the transverse direction. Simply put, the most significant dissimilarity between children without a cleft and those with UCLP treated with a one-stage method pertained to the width of the palatal vault, which was narrower in the cleft groups. Our results are in partial agreement with the findings of Rusková et al. [31], who also used geometric morphometrics to analyze palatal shape in individuals with UCLP. However, in the latter study, palatal shape was assessed at an average age of 15 years (ranging from 12 to 17 years). The authors found that the average UCLP palate was shallower, narrower, shorter, and more asymmetrical compared to controls despite prolonged and intensive orthodontic treatment. The degree of dysmorphology appears to be significantly greater in their cleft sample than in ours. This difference might be attributed to the age at the evaluation, as we assessed patients several years younger, but it may also reflect suboptimal treatment outcomes in the sample studied by Rusková et al. [31], as indicated by significant palatal scarring noticeable in the illustration of the cleft palate. However, it is important to note that the patients evaluated by Rusková et al. [31] had undergone surgery between 1975 and 1980, before the popularization of surgical techniques aiming to reduce scar formation.

The disparity between our results and those of investigations involving linear, area, and volumetric assessments by Bittencourt et al. [32] and Generali et al. [33] is also noteworthy. These studies reported maxillary arch constriction in the cleft area and width similarity in the molar region in preadolescent patients with UCLP compared to controls. However, our study suggests that the intermolar width in the current cleft sample is wider than in controls, as indicated by the superimposition of consensus palatal shapes. One possible explanation for this discrepancy is that our patients received orthodontic treatment with expansion plates, which could have contributed to increased maxillary arch dimensions.

In our study, there was a variation in the average age at which we assessed palatal shape among the different groups. The group without a cleft condition consisted of the youngest subjects, with an average age of 8.6 years. On the other hand, patients with a cleft were around 1 year older in the case of early ABG and approximately 2 years older for those with late ABG compared to the control group. Although the assessment age was before the growth spurt, we acknowledge that developmental changes during this period might have potentially influenced the results. However, the findings of studies that have evaluated palatal vault growth in non-cleft children [34,35] have indicated that changes in palatal dimensions between 8 and 10 years of age are limited. For instance, Yang et al. conducted biannual measurements of palatal widths and heights from 6 to 14 years of age and found that yearly changes were generally less than 0.5 mm. The intercanine width increased by 0.3 mm from 8 to 10 years, and the intermolar distance increased by 0.6 mm. Palatal height showed an increase of 0.2 to 0.9 mm (on average less than 0.5 mm) over 2 years, depending on the place of measurement. Considering these findings, it is reasonable to conclude that the differences in the age of assessment among our study groups likely had a negligible effect on our results.

As highlighted in the introduction, the advantages of performing ABG between 2 and 4 years of age can encompass various benefits. These include the potential for early closure of oro-nasal fistulas [36,37], improved support for the movement and positioning of the tongue and lips during speech development, which may contribute to enhanced speech articulation [38,39], and a potential decrease in the need for additional surgical interventions in later stages of treatment, promoting a more streamlined and less invasive overall treatment. However, it is important to exercise caution when drawing conclusions about the timing of ABG used in our center, considering the absence of reports from other cleft centers employing a comparable timing. Definitive confirmation of the efficacy of our approach can only be established through a comprehensive assessment of the outcomes of our entire treatment protocol in early adulthood when growth is completed [40,41,42,43,44,45].

## 5. Limitations

This study has several limitations that should be considered when interpreting the results.

Firstly, this is a single-center study using a specific treatment protocol performed by five experienced, high-volume surgeons. Therefore, the generalizability can be limited. Secondly, this is a retrospective study, and patients in the cleft groups were not operated consecutively. This non-consecutive patient inclusion could potentially introduce a selection bias, as it may not accurately represent the entire population of subjects with a cleft. To enhance the generalizability of the findings, future studies should endeavor to include consecutive patients. An additional limitation to consider is the margin of error in our methodology, which contributed to 10.8% of the overall shape variation. This margin of error is roughly 40% higher than what has been observed in earlier investigations that examined palatal shape. It is important to note, however, that previous studies predominantly focused on individuals without cleft conditions, who tend to exhibit a more consistent palatal morphology. In contrast, the presence of cleft-related deformities can introduce additional complexity into the analysis.

## 6. Conclusions

The findings of this study suggest that alveolar bone grafting performed at approximately 2 years of age has a limited negative effect on palatal morphology. Based on palatal shape as an indicator of future maxillary development, it is reasonable to anticipate similar growth outcomes in patients who underwent early ABG compared to those who received ABG within the conventional timeframe of 9–11 years. However, to fully understand the implications of early ABG on maxillary growth and overall craniofacial development, further research is essential. Long-term follow-up studies with larger patient cohorts are warranted to explore the functional consequences of palatal shape differences and to assess the comprehensive effects of early ABG.

## Figures and Tables

**Table 1:** Demographic description of the groups.

	Age at Cleft Repair (Yrs.) Mean (SD), [min–max]	Age at ABG (Yrs.) Mean (SD), [min–max]	Age at Collection of Models (Yrs.) Mean (SD), [min–max]
**Early ABG**			
males (*n* = 21)	0.6 (0.1); [0.4–0.8]	2.1 (0.6); [1.4–4.1]	9.5 (0.8); [8–11.3]
females (*n* = 7)	0.7 (0.2); [0.5–1.1]	2.1 (0.5); [1.6–3.1]	9.8 (0.4); [8.8–10.1]
males and females (*n* = 28)	0.6 (0.1); [0.4–1.1]	2.1 (0.6); [1.4–4.1]	9.5 (0.7); [8–11.3]
**Late ABG**			
males (*n* = 19)	0.8 (0.2); [0.5–1.2]	8.5 (1.2); [7.3–12.8]	11.3 (1.5); [9.5–13.9]
females (*n*= 14)	0.7 (0.1); [0.5–1]	8.7 (1.4); [7.2–12.9]	10.1 (1.3); [8–13.9]
males and females (*n* = 33)	0.7 (0.2); [0.5–1.2]	8.6 (1.3); [7.2–12.9]	10.8; 1.5; [8–13.9]
**Control**			
males (*n* = 25)	N/A	N/A	8.5 (1); [6.8–11.6]
females (*n* = 35)	N/A	N/A	8.6 (1.4); [6.3–11.8]
males and females (*n* = 60)	N/A	N/A	8.6 (1.2); [6.3–11.8]

N/A—not applicable.

**Table 2:** Non-trivial principal components in the whole sample.

	% Variance	% Cumulative Variance	Broken-Stick Criterion	Variance
PC1	26.1%	26.1%	1.0%	0.003247
PC2	12.0%	38.1%	0.9%	0.001487
PC3	11.9%	49.9%	0.8%	0.001480
PC4	7.8%	57.7%	0.7%	0.000967
PC5	6.4%	64.1%	0.7%	0.000799
PC6	4.7%	68.8%	0.7%	0.000584
PC7	3.7%	72.6%	0.7%	0.000465
PC8	3.6%	76.1%	0.6%	0.000444
PC9	2.9%	79.1%	0.6%	0.000365
PC10	2.4%	81.4%	0.6%	0.000296
PC11	2.0%	83.4%	0.6%	0.000244
PC12	1.6%	85.0%	0.6%	0.000194
PC13	1.3%	86.2%	0.6%	0.000157
PC14	1.1%	87.3%	0.6%	0.000137
PC15	1.0%	88.3%	0.5%	0.000119
PC16	0.9%	89.2%	0.5%	0.000110
PC17	0.8%	90.0%	0.5%	0.000098
PC18	0.7%	90.7%	0.5%	0.000093
PC19	0.7%	91.4%	0.5%	0.000083
PC20	0.6%	91.9%	0.5%	0.000070
PC21	0.5%	92.4%	0.5%	0.000062
PC22	0.5%	92.9%	0.5%	0.000057

**Table 3:** Intergroup differences in shape space.

	Difference in Procrustes Distance	*p*-Value
Control vs. Early ABG	0.0951	<0.001
Control vs. Late ABG	0.0967	<0.001
Early vs. Late ABG	0.0407	0.042

## Data Availability

The data is contained within the article.
